# A new proposal for the use of the focal animal technique in buffaloes in the Eastern Amazon

**DOI:** 10.3389/fvets.2023.1266451

**Published:** 2023-11-08

**Authors:** Welligton Conceição da Silva, Jamile Andréa Rodrigues da Silva, Amauri Gouveia Júnior, Adriano Braga Brasileiro de Alvarenga, Antônio Vinícius Correa Barbosa, Éder Bruno Rebelo da Silva, Maria Roseane Pereira dos Santos, José de Brito Lourenço-Júnior, Raimundo Nonato Colares Camargo Júnior, André Guimarães Maciel e Silva

**Affiliations:** ^1^Postgraduate Program in Animal Science (PPGCAN), Institute of Veterinary Medicine, Federal University of Para (UFPA), Federal Rural University of the Amazônia (UFRA), Brazilian Agricultural Research Corporation (EMBRAPA), Castanhal, Brazil; ^2^Institute of Animal Health and Production, Federal Rural University of the Amazônia (UFRA), Belém, Brazil; ^3^Laboratory of Neuroscience and Behavior, Federal University of Pará (UFPA), Belém, Brazil; ^4^Department of Animal Sciences, University of Brasília, Brasília, Brazil; ^5^Institute of Veterinary Medicine, Federal University of Pará (UFPA), Castanhal, Pará, Brazil; ^6^Cyberspace Institute, Federal Rural University of the Amazônia (UFRA), Belém, Brazil; ^7^Institute of Engineering and Geosciences, Federal University of Western Pará (UFOPA), Santarém, Brazil

**Keywords:** observation technique, animal behavior, snapshot recording, buffalo, ethogram

## Abstract

This study aims to present a proposal for using the focal animal recording technique to evaluate the welfare of buffaloes and to verify the association between each behavior and thermal comfort indices. The study was conducted in an experimental paddock located in Santarém, Pará, Brazil. A total of 10 female Murrah animals were used. The behavior of the animals was recorded during the day, with the use of three trained observers, for 72 consecutive h. Climatic variables were collected, and the Temperature–Humidity Index (THI) and the practical Buffalo Comfort Climatic Conditions Index (BCCCIp) were determined. The multivariate technique of principal components and Spearman’s correlation were employed. BCCCIp and THI were outside the thermal comfort zone at different times of the day. Grazing (P) was more frequent in the coldest hours of the day, while rumination occurred at different periods, mainly during the daytime and frequently in a lying position. There was a positive correlation between idle lying behavior and average temperature—Tmed (*r* = 0.583; *p* < 0.028), THI (*r* = 0.432; *p* < 0.034), and BCCCIp (*r* = 0.554; *p* < 0.049). There was a positive correlation between grazing and Tmed (*r* = 0.665; *p* < 0.0004) and BCCCIp (*r* = 0.583; *p* < 0.036). The standing idle behavior negatively correlated with Tmed (*r* = −0.718; *p* < 0.0001), THI (*r* = −0.522; *p* < 0.008), and BCCCIp (*r* = −0.8076; *p* < 0.0008). The lying ruminating behavior had a positive correlation with Tmed (*r* = 0.586; *p* < 0.002), THI (*r* = 0.477; *p* < 0.018), and BCCCIp (*r* = 0.8033; *p* < 0.0009). Furthermore, ruminating while standing correlated positively with Tmed (*r* = 0.680; *p* < 0.0003). The adaptation of the focal animal technique, with six observers evaluating each animal for 6 min through filming, proved to be efficient in pointing out the different behaviors of buffalo raised in Eastern Amazon fields under heat stress at different times of the day.

## 1. Introduction

The focal animal method described by Altmann (1) involves recording the behavior of an individual or a group of animals. For this method to be effective, a predetermined observation time to observe the sample subject must be set and recorded for each session. The length of each sampling session depends on the observer’s fatigue and physical readiness. This information is important to determine the appropriate experimental methodology.

The focal animal technique is a cost-effective and less laborious approach to identifying behavioral parameters. It requires fewer animals and observations to represent the entire group. The former factor helps to identify abnormal behaviors both inside and outside the natural environment, such as in pastures. This method is useful in recognizing any unusual behavior patterns (1).

Some studies were conducted to identify the behavior of cattle in different environments, such as in the intervals between milking dairy cows (2–4) or in the field (5), which contributes to the proper management of individuals by favoring animal welfare (AW) and enhancing herd productivity (6–10).

Different studies were carried out to observe the behavior in different environments, including their access to shade and water for immersion. The results showed that the buffaloes preferred to stay protected from solar radiation during the hottest hours of the day and that they used water as their preferred environment for cooling. For example, water buffaloes were observed grazing in Lake Kerkini National Park in northern Greece (11).

In general, observations are carried out by taking into account different behaviors that could signal the degree of AW in cattle, pigs, buffaloes, sheep, and goats. When coded correctly, these behaviors can provide information that will help in the identification of abnormalities, with the goal of adopting management techniques that favor the reduction of the expression of undesirable behaviors to favor AW.

In this context, some indices help to determine the influences resulting from these production systems, such as the Temperature–Humidity Index (THI) and the Practical Buffalo Comfort Climatic Conditions Index (BCCCIp), calculated according to meteorological variables and physiological parameters of buffaloes (2), signaling stress in animals (6, 7).

Based on this information, this study aims to present a proposal for using the focal animal recording technique to evaluate the welfare of buffaloes and to verify the associations between each behavior and thermal comfort indices. The animals were kept in an experimental paddock in Santarém, Pará, Brazil.

## 2. Materials and methods

### 2.1. Observation environment

**Figure 1:**
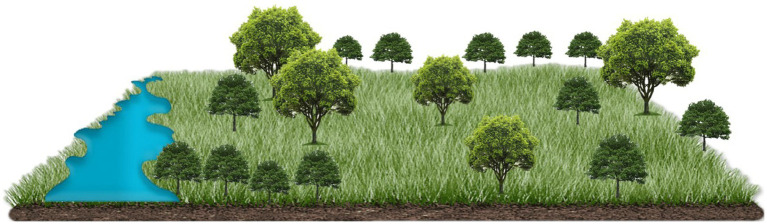
The study site’s infographic shows the distribution of trees within the rural property.

The experiment took place in a paddock of rural property in Santarém, Pará, Brazil, with an area of 2.5 ha of *Brachiaria decumbens*, with trees homogeneously distributed for natural shadings, such as Jatobá (*Hymenaea courbaril*), with approximately 10 m in height, and other species, with a height of less than 5 m, with a lake of 50 m wide and 2 m deep, and with free access given to the animals for immersion and a source of hydration for both the individuals and the animals (Figure 1).

### 2.2. Experimental animals

From a group of 15 buffaloes, 10 clinically healthy, non-lactating Murrah females were selected, with an average weight of 550 ± 26 kg and an average age of 24 ± 2.3 months who had lived together for more than a year. The paddock capacity was 7.33 animal units (AU)/hectare.

### 2.3. Ethogram

The animals were filmed during the day using a cell phone video camera, Sony®, model: Cyber-shot, DSC-TF1, resolution: 16.1 MP. Filming was carried out during both the day and night in three different periods, specifically in the morning from 6:00 a.m. to 9:00 a.m., midday from 9:01 a.m. to 2:00 p.m., and in the afternoon from 2:01 p.m. to 6:00 p.m. The animals were given a 5-day adaptation period to get used to the presence of the observers. The camera was positioned approximately 3 m away from the animals; however, this variation may be greater due to the animals walking in the paddock, which did not prevent the animals from being viewed.

Observations were carried out over 3 consecutive days. Each focal animal was observed every hour for 6 min per hour. This time was adopted, as 6 min per animal was defined since an hour has 60 min. Consequently, these were equally distributed among the 10 experimental animals (60 min/10 animals = 6 min/animal). In total, six specialized observers alternated every 6 min per hour to reduce possible influences on confirming behavior.

In the analysis of the footage, the images were transferred to the computer, and three trained observers analyzed them separately using Cowlog 3.0 software (12).

The predefined behavioral variables, according to the literature, were posture (standing or lying down), activities performed (grazing, rumination, and idleness) (state type), tail wagging, scratching, and licking (instant) (Table 1), with the state type described as behaviors greater than 5 s and instantaneous (less than 5 s), as described by Lehner (15). It was also observed whether the animals were immersed in the lake to cool off. The number of times the behavior was performed in a given period of time was recorded.

**Table 1:** Ethogram: pre-defined behaviors according to the literature and its definitions.

Behavior	Definition
Standing	Leaning on its limbs, moving or standing still.
Lying down	Animal with four-flexed legs and with the abdomen wholly or partially in contact with the earth.
Grazing	Act of feeding on pasture, always on foot.
Ruminating	Animal chewing, swallowing, regurgitating, and re-chewing with the presence of bolus
Idleness	Looking in any direction without visible appearance, lying, or standing.
Wagging the tail	Flank of the cheek, which can be performed
Scratching the body	Standing or lying down.
Licking	Looking inattentively in any direction

Source: Adapted from Coimbra et al. (13), Agudelo et al. (14), and Silva et al. (4).

### 2.4. Climate variables

The climatic variables, including air temperature (AT), relative humidity (RH), and dew point, were measured using a thermo-hygrometer, Incoterm® 5203.03.0.00. The device was installed at a fixed wooden point in the paddock in which the animals were conditioned, and the data were recorded every 15 min, and these data were presented in the tables on average every hour.

### 2.5. Temperature–Humidity Index (THI)

The THI was calculated by taking into account temperature and relative humidity. The THI value up to 72 was considered thermal comfort; between 72 and 78 signals mild or mild thermal stress; between 79 and 88 signals moderate thermal stress; and between 89 and 98 signals severe stress (16).

THI was obtained through Equation 1, adapted from Thom (16).

$THI=\left(0.8T\right)+\left(RH/100\right)*\left[\left(T-14.4\right)+46.4\right]$

where T is the dry bulb air temperature (°C) and RH is the relative air humidity (%).

### 2.6. Practical Buffalo Comfort Climatic Condition Index (BCCCIp)

The practical Buffalo Comfort Climatic Conditions Index (BCCCIp) was calculated by taking into account the temperature and relative humidity of the air. The following bands were considered: —comfort (≤ 34.65), danger (34.66–38.02), stress (38.03–41.39), and emergency (≥ 41.40) (17).

The BCCCIp was calculated using the formula:

BCCCIp = 0.0571*RH + 1.0480*AT,

where RH is the relative humidity of the air (%) and AT is the air temperature (°C).

### 2.7. Statistical analysis

With the objective of evaluating the behavior of the animals throughout the shifts of the day and facilitating the understanding of the behaviors (morning from 6:00 a.m. to 9:59 a.m., midday from 10:00 a.m. to 1:59 p.m. and afternoon from 2:00 p.m. to 6:00 p.m.), the multivariate technique of principal components was used to group the behaviors and to identify which activities the animals perform most in these periods. In addition, the behaviors were also evaluated using Spearman’s correlation (at a significance level of 5%) with the values of THI, Tmed, and BCCCIp to identify positive and negative correlations that may influence the activities that the animals perform throughout the day. Data were also initially analyzed by comparing the activities in each shift for both sun and shade treatments using the chi-squared test and a subsequent comparison of pairs of values using the chi-squared test with Bonferroni correction. Subsequently, the sun and shade treatments were compared for each shift using the Wilcoxon test. All analyses consider a significance level of 5% and were performed using the R-Studio software, version 1.1.463 3 (18).

## 3. Results and discussion

When analyzing the trends in average air temperature over 3 days, it became evident that temperatures were consistently higher during the day. This was evidenced by the temperature readings that showed 30.2°C in the morning and 32.5°C in the afternoon. However, 27.5°C was observed at night (as indicated in Table 2). The temperatures considered favorable for breeding (including reproductive and productive aspects) of buffaloes ranged from 13°C to 18°C; thus, the animals were able to express their normal characteristics and well-being on pasture (19).

**Table 2:** Meteorological indices and animal thermal comfort of buffalo raised in the paddock with the availability of shade in Santarém-PA, Brazil.

Period	Time	AT (°C)	THI	BCCCIp
Morning	06:00	27.5	79.8	33.27
	07:00	27.5	79.8	33.01
	08:00	27.2	79.6	32.80
	09:00	27.5	79.8	33.37
Midday	10:00	28	80.8	33.47
	11:00	32	86.3	34.69
	12:00	30.2	83.7	36.07
	13:00	32	86.3	37.79
Afternoon	14:00	31.7	79.2	37.66
	15:00	32.5	87.3	38.03
	16:00	31.3	79.8	35.06
	17:00	28.5	81.8	33.73
	18:00	26	77.5	32.16

AT, Average temperature; THI, Temperature–Humidity Index; BCCCIp, practical Buffalo Comfort Climatic Conditions Index (17).

During the intermediate period between 1:00 p.m. and 3:00 p.m., the highest temperature, THI, and BCCCIp were recorded, which could cause heat stress in animals (20). Animals develop heat stress due to an imbalance between high environmental temperatures and metabolic heat production. Furthermore, when thermoregulatory mechanisms such as behavioral, endocrine, and metabolic responses are not capable of restoring thermoneutrality or when they are not sufficient, heat stress can be triggered. By including this at the beginning of the Discussion section, the reader will be able to easily understand why all the variables evaluated in the present study are associated (21, 22). Therefore, environmental variables have a direct influence on the thermoregulation of buffaloes, which can lead to problems in growth and reproduction (23, 24).

The temperatures mentioned above were observed during the study of buffalo behavior. It is an important factor to consider as buffaloes have peculiar characteristics, such as a low number of sweat glands and a high concentration of melanin. This makes them more susceptible to heat and solar radiation (25–27) and heat stress, causing them to spend more time grazing when temperatures are lower (28–30).

The relative humidity of the air was high at some hours of the day, reaching values between 87 and 100%. According to Azevedo and Alves (31), animals begin to express their productive potential when the humidity levels are between 60 and 70%, which are considered adequate for the species.

The THI determines and quantifies the intensity of heat stress in domestic species (8). Thus, it was observed that the THI values for buffalo breeding were higher than the recommended values (32). For the animals to be considered within the thermoneutrality zone, the THI must remain up to 72. If the THI is between 72 and 78, it indicates mild stress or mild thermal stress. If the THI ranges between 79 and 88, it suggests moderate stress, and between 89 and 98, it indicates severe stress. Similar results were reported in the study by Moraes et al. (33), Pantoja et al. (34), and Almeida et al. (30).

The BCCCIp, which is used for diagnosing cases of heat stress in buffalo herds, indicated that between 12:00 h and 13:00 h, the buffaloes were in the danger zone (34.66–38.02), and at 15:00 h they were under heat stress (38.03–41.39) (17). This may be related to the environment, as the behavior and physiology of buffaloes have a direct influence on this variable, resulting in negative effects on animal welfare (6, 35–37).

**Figure 2:**
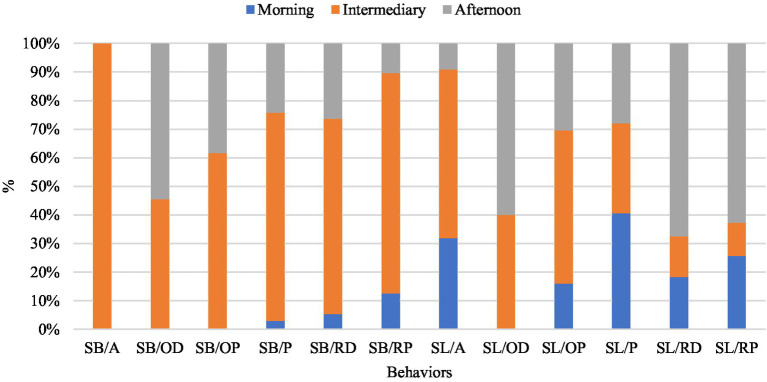
Behavior of buffaloes raised on pasture, in sun and shade, in the Eastern Amazon. SB/A, Shadow walking; SB/OD, Lying Idle Shadow; SB/OP, Standing Idle Shadow; SB/P, Shadow Grazing; SB/RD, Shadow Ruminating Lying Down; SB/RP, Standing Ruminating Shadow; SL/A, Walking Sun; SL/OD, Sun in Leisure Lying; SL/OP, Standing Idle Sun; SL/P, Sun grazing; SL/RD, Sun ruminating lying down; SL/RP, Sun ruminating standing.

The most expressive behavior observed in buffaloes raised on pasture was grazing, followed by rumination and minimal idling (Figure 2). The low idleness rate may be due to good-quality pasture, which provides the animals with a greater ability to perform the activity (38). In a study evaluating behavioral responses relating to the thermoregulation capacity of buffaloes, grazing was the most frequent behavior observed, regardless of access to water for immersion (11).

In relation to the morning shift, it was noted that the animals graze in a shaded area, possibly due to stress from high temperatures and intense solar radiation (26, 37). These results support the findings of Almeida et al. (30), who found no significant differences; that is, the presence of shading did not interfere with the grazing of the animals. This is likely due to the colder air temperature during the experiment.

Rumination was more frequent during the hottest hours of the day, mainly in the lying position and in a shady environment. This is possibly due to the fact that the animals sought shade to cool off and eat more during the night when temperatures were lower. During the day, when temperatures are high, animals tend to ruminate on food consumed at night. With similar results, Santos et al. (38), in the Amazon, observed that buffalo heifers sought shaded areas, especially during the hottest hours of the day, to ruminate, both standing and lying down, in search of a more suitable place for their well-being.

The standing and lying positions were evidenced according to the behavior or activity that the individual performed: the first position, standing, was observed in most situations, mainly during grazing; however, the second position, lying, was less adopted by buffaloes while ruminating, mostly in the shade. This fact was observed in studies by Santos et al. (39). It is noteworthy that this position is more commonly used in the rumination process. Corroborating the studies carried out by Ablas et al. (11) and Marques et al. (40), it was identified that the lying position was the most used position by animals in the rumination process.

The rumination time was lower than the grazing time in all shifts; however, rumination is more common in warmer periods of the day, being carried out mainly in a lying position and in shaded areas (*p* < 0.05) (Table 3), as the canopy of trees prevents the exacerbated passage of solar radiation, promoting a reduction in the temperature of 2 to 3°C when compared to places without trees. With this decrease in temperature, there is a reduction of heat in the animals, which consequently provides them with a more thermally comfortable environment (41, 42).

**Table 3:** Behavior of buffaloes raised on pasture, in sun and shade, in the Eastern Amazon.

Shadow (Minute)						
Shift	A	OD	OP	P	RD	RP
Morning	0a	0a	0a	6b	30c	6b
Midday	10a	5a	45b	150c	389d	37b
Afternoon	0a	6b	28c	50d	150e	5b

Sun (Minute)						
Shift	A	OD	OP	P	RD	RP
Morning	21b	0a	11b	1246d	59c	11b
Midday	39b	2a	37b	968c	46b	5a
Afternoon	6a	3a	21b	860d	218c	27b

A, walking; OD, leisurely lying down; OP, idle standing; P, grazing; RD, ruminating lying down; RP, ruminating while standing. Different letters (a, b, c, d) in the line indicate statistical differences (*p* < 0.05).

During the morning shift, the predominant behavior of the animals was grazing in the sun. As for the afternoon shift, the primary activities observed were animals ruminating while standing and lying down in sunny areas. During the midday shift, activities such as walking, grazing, and ruminating while standing and lying down were notably observed in the shaded areas.

The animals spend most of their time in a lying position, showing a good AW index (43, 44).

The behavior of lying down in buffaloes is associated with thermoregulation because lying on a cold surface can promote heat dissipation by convection, just as lying down reduces movement and, therefore, reduces the amount of heat produced by the body (6).

There was a positive correlation between lying down and AT (*r* = 0.583; *p* < 0.028), THI (*r* = 0.432; *p* < 0.034), and BCCCIp (*r* = 0.554; *p* < 0.049) (Table 4). This occurs because the hottest hours of the day are used to practice this activity, usually in the shade, avoiding an increase in body temperature, which is a thermoregulatory behavior of buffaloes (27, 33). Research evaluating the thermoregulatory behavior of buffaloes indicates that shade is an important component to help dissipate heat under different conditions (6, 37, 38, 45, 46). Furthermore, heat stress tends to reduce grazing activity (47).

**Table 4:** Positive and negative correlations between the variables, average temperature, THI, and BCCCIp, were equally observed in all shifts.

Behaviors	R2 strength and *p* value	Indices		
		AT	THI	BCCCIp
A	R^2^	0.42979	0.36827	0.48262
	*p* value	0.1427	0.2157	0.0948
OD	R^2^	0.64498	0.56337	0.55479
	*p* value	0.0173	0.0450	0.0491
P	R^2^	0.63436	0.51262	0.58322
	*p* value	0.0199	0.0733	0.0364
OP	R^2^	−0.87692	−0.68348	−0.80769
	*p* value	<0.0001	0.0100	0.0008
RD	R^2^	0.77841	0.57268	0.80330
	*p* value	0.0017	0.0408	0.0009
RP	R^2^	−0.07961	−0.14789	−0.21083
	*p* value	0.7960	0.6297	0.4893

A, walking; OD, idle lying down; P, grazing; OP, idle standing; RD, ruminating lying down; RP, ruminating standing up; AT, average temperature; THI, Temperature–Humidity Index; BCCCIp, practical Buffalo Comfort Climatic Condition Index. A value of *p* of < 0.05 indicates a statistical difference.

There was a positive correlation between grazing and AT (*r* = 0.665; *p* < 0.0004) and BCCCIp (*r* = 0.583; *p* < 0.036). This indicates that when the temperature increases, grazing also tends to increase; however, in general, this happens in shaded areas (37, 48), and, as previously shown, this is a common practice in the morning shift.

Standing idle behavior was negatively correlated with AT (*r* = −0.718; *p* < 0.0001), THI (*r* = −0.522; *p* < 0.008), and BCCCIp (*r* = −0.8076; *p* < 0.0008). This can possibly be explained as a result of this activity being performed when the animals seek to reduce body heat, such as foraging, ingesting and digesting food, and absorbing nutrients, which are processes that generate heat. In addition, therefore, reducing food consumption provides thermoregulation (49). Thus, heat stress stimulates peripheral heat receptors to transmit suppressor nerve impulses to the appetite center present in the hypothalamus, causing a decrease in food consumption (50) and consequently performing idle behavior. Similar results on the reduction of food intake during heat stress were evidenced in the studies by Habeeb et al. (51), Khongdee et al. (52), and Savsani et al. (53).

The lying ruminating behavior had a positive correlation with AT (*r* = 0.586; *p* < 0.002), THI (*r* = 0.477; *p* < 0.018), and BCCCIp (*r* = 0.8033; *p* < 0.0009). Furthermore, ruminating while standing correlated positively with AT (*r* = 0.680; *p* < 0.0003). This fact stems from this activity being carried out in the shade, providing animal well-being, as well as because this is an evolutionary adaptation of buffaloes that express their thermoregulatory behavior lying under the shade of trees (52). In this situation, we recommend taking wind speed into account, as other studies used fans in the summer and, for this reason, identified a greater adoption of the lying position in buffaloes, as described in the studies by Ahmad et al. (54).

The animals spent an average of 3 h in the lake, usually in the afternoon shift from 1 p.m. to 3 p.m. Buffaloes have only one-sixth of sweat glands (55) and fewer hairs than cattle (56, 57). In addition, buffaloes have difficulty thermoregulating at high temperatures due to their dark skin and thick epidermis (58), which can often present lesions depending on the region of the country (59), where the presence of solar radiation can be more intense. Thus, these individuals suffer from thermal stress when exposed to solar radiation and consequently immerse themselves in water to minimize stress under these environmental conditions (37) or even tend to ingest more water (60–62). Water intake by buffaloes is linked to dehydration due to massive sweating, aiming to maintain the thermoregulation that induces the thirst center in the hypothalamus during excessive heat load (61).

Note that tail wagging was routinely observed (721 times), differing from the other behaviors, in the period of study in hours, and each movement lasted a maximum of 5 s (Table 5), according to the methodology of Almeida et al. (63). This can be considered an important parameter, which demonstrates a degree of AW classified as good, if we consider that the environment had leaking places, such as, for example, shade and water (64). However, if we observe the CI and THI, these are above the reference value, which can cause heat stress but is generally mitigated by the presence of trees. Corroborating with Albright and Arave (65), it was revealed that tail movements observed in cattle were reported as a strong indicator of mood and well-being.

**Table 5:** List of instantaneous behaviors observed in buffaloes grazing in the municipality of Santarém, Pará, Brazil.

Behavior	Quantity (movements)	Duration (amount of time per behavior)
Wagging the tail	721a	3–5 s
Scratching the body	142b	15–30 s
Licking	82c	30–37 s

Different letters in column (a, b) indicate statistical differences (*p* < 0.05 – chi-squared test).

Scratching body parts using a limb was recorded 142 times, lasting from 15 to 37 s per execution. Licking one’s own body or that of another animal was an uncommon practice among buffaloes, observed only 82 times with a duration of 30–37 s per behavior. Scratching and licking are commonly identified in the relationship between mother and calf; however, as observed in this study, these behaviors are positive and are observed in buffaloes over 20 months of age and not restricted to maternal coexistence. Scratching and licking are presented as expressions of social behavior when practiced through interaction between animals and are considered positive and/or appropriate behaviors for dairy buffaloes (66).

However, interpretations of this behavior must be considered, particularly when associating this behavior with others. Degasperi et al. (66), when studying the behavior of cattle, found that the practice of licking oneself or another animal is a behavior inherited from wild ancestors, which had a function linked to cleaning and extracting salt from the animals’ fur. According to the author, domestic cattle practice this behavior, which is relevant to social dynamics and hygiene, and another factor linked to this behavior is physiological, which signals a reduction in heart rate, giving a sense of calmness.

In this way, this study provides insights into the use of focal animal recording techniques to assess the welfare of buffaloes and their association with thermal comfort indices. The results have several implications for future research and practical applications:

1. Improved welfare assessment: Focal animal recording techniques have proven to be effective in capturing and analyzing buffalo behavior under different climatic conditions. This approach offers a more detailed and complex understanding of buffalo welfare, enabling more informed management practices.
2. Identification of thermal stress patterns: The study’s results highlight the relationship between buffalo behavior and thermal comfort indices (THI and BCCCIp). This information can be crucial for identifying specific patterns of thermal stress and discomfort in buffaloes and guiding the development of targeted interventions.
3. Applicability to different climate zones: The success of the study in the Eastern Amazon region, which experiences thermal stress at different times of the day, suggests that focal animal recording techniques can be adopted and applied in various climatic conditions, making it a valuable tool for assessing buffalo welfare in different geographical regions.
4. Enhanced animal management: By understanding how buffalo behavior correlates with environmental factors such as temperature and humidity, farmers and animal welfare professionals can implement more effective management strategies. For example, adjusting feeding and resting schedules based on temperature fluctuations can help improve buffalo comfort and productivity.
5. Directions for future research: This study opens avenues for further research into buffalo welfare and the mitigation of thermal stress. Future studies can build upon these results to develop real-time monitoring systems or decision-support tools that assist farmers in making timely interventions to ensure the well-being of their buffalo herds.

## 4. Conclusion

The adaptation of the focal animal technique, with six observers evaluating each animal for 6 min through filming, proved to be efficient in identifying different behaviors of buffaloes raised in the fields of the Eastern Amazon under heat stress at different times of the day. Furthermore, it is currently possible to highlight a variation in behavior between morning and afternoon shifts, with grazing being more prevalent in the morning and rumination in shaded areas being more common in the afternoon. Therefore, the technique adequately reflects the behavioral repertoire of the species.

## Data availability statement

The raw data supporting the conclusions of this article will be made available by the authors, without undue reservation.

## Ethics statement

The experimental procedures were carried out in accordance with Brazilian law and were previously approved by the Animal Ethics Committee (CEUA/UFPA), under protocol number 9307300720. The study was conducted in accordance with the local legislation and institutional requirements.

## Author contributions

WS: Conceptualization, Data curation, Formal analysis, Funding acquisition, Investigation, Methodology, Project administration, Resources, Software, Writing – review & editing. JS: Investigation, Methodology, Writing – review & editing. AG: Investigation, Methodology, Writing – original draft. AA: Investigation, Methodology, Writing – original draft. AB: Investigation, Methodology, Writing – original draft. ÉS: Investigation, Methodology, Writing – original draft. MP: Investigation, Methodology, Writing – original draft. JL-J: Investigation, Methodology, Writing – original draft. RC: Investigation, Methodology, Writing – original draft. AS: Conceptualization, Formal analysis, Funding acquisition, Investigation, Methodology, Resources, Writing – original draft.

## References

[1] Altmann J. Observational study of behavior: sampling methods. Behaviour. (1974) 49:227–67. doi: 10.1163/156853974X00534, PMID: 4597405

[2] Chikkagoudara KP Singh P Bhatt N Barman D Selvaraj R Lathwal SS. Effect of heat stress mitigations on physiological, behavioural, and hormonal responses of Buffalo calves. Int J Biometeorol. (2022) 66:995–1003. doi: 10.1007/s00484-022-02255-9, PMID: 35124759

[3] Petrocchi Jasinski F Evangelista C Basiricò L Bernabucci U. Responses of Dairy Buffalo to Heat Stress Conditions and Mitigation Strategies: A Review. Animals. (2023) 13:1260. doi: 10.3390/ani13071260, PMID: 37048516 PMC10093017

[4] Silva WCD Silva ÉBRD Santos MRPD Camargo Junior RNC Barbosa AVC Silva JARD. Behavior and thermal comfort of light and dark coat dairy cows in the Eastern Amazon. Front Vet Sci. (2022) 9:1006093. doi: 10.3389/fvets.2022.1006093 36187817 PMC9516290

[5] Pereira KCB Carvalho CDCS Ruas JRM Menezes GCDC Castro ALDO Costa MDD. Effect of the climatic environment on ingestive behavior of F1 Holstein x Zebu cows. Rev Bras Saúde Prod Anim. (2018) 19:207–15. doi: 10.1590/s1519-99402018000200006

[6] Silva JARD Pantoja MHDA Silva WCD Almeida JCFD Noronha RDPP Barbosa AVC. Thermoregulatory reactions of female buffaloes raised in the sun and in the shade, in the climatic conditions of the rainy season of the Island of Marajó, Pará, Brazil. Front Vet Sci. (2022) 9:998544. doi: 10.3389/fvets.2022.1031330, PMID: 36176704 PMC9513356

[7] Silva WCD Silva JARD Camargo-Júnior RNC Silva ÉBRD Santos MRPD Viana RB. Animal welfare and effects of per-female stress on male and cattle reproduction—A review. Front Vet Sci. (2023) 10:1083469. doi: 10.3389/fvets.2023.1083469 37065229 PMC10102491

[8] da Silva WC Printes OVN Lima DO da Silva ÉBR dos Santos MRP Júnior RNCC. Evaluation of the temperature and humidity index to support the implementation of a rearing system for ruminants in the Western Amazon. Front Vet Sci. (2023) 10:8678. doi: 10.3389/fvets.2023.1198678 PMC10375705 37520006

[9] Mota-Rojas D Titto CG Orihuela A Martínez-Burnes J Gómez-Prado J Torres-Bernal F. Physiological and behavioral mechanisms of thermoregulation in mammals. Animals. (2021) 11:1733. doi: 10.3390/ani11061733, PMID: 34200650 PMC8227286

[10] Mota-Rojas D Napolitano F Braghieri A Guerrero-Legarreta I Bertoni A Martínez-Burnes J. Thermal biology in river buffalo in the humid tropics: Neurophysiological and behavioral responses assessed by infrared thermography. J Anim Behav Biometeorol. (2020) 9:2103. doi: 10.31893/jabb.21003

[11] Tsiobani ET Yiakoulaki MD Hasanagas ND Menexes G Papanikolaou K. Water buphaloes grazing behaviour at the Lake KerkiniNational Park, Northern Greece. Hacquetia. (2016) 15:133–42. doi: 10.1515/hacq-2016-0015, PMID: 35879541

[12] Hänninen L Pastell M. CowLog: Open source software for coding behaviors from digital video. Behav Res Methods. (2009) 41:472–6. doi: 10.3758/BRM.41.2.472, PMID: 19363187

[13] Coimbra PAD Machado Filho LCP Hötzel MJ. Effects of social dominance, water trough location and shade availability on drinking behaviour of cows on pasture. Appl Anim Behav Sci. (2012) 139:175–82. doi: 10.1016/j.applanim.2012.04.009

[14] Agudelo JAB Agudelo SAF Quadros LCP. Coçar, limpar e formar laços sociais: aliciamento e seu significado biológico em ruminantes. CES Medicina Veterinaria e Zootecnia. (2013) 8:120–31.

[15] Lehner PN. Hand book of ethological methods. New York: Garland STPM Press (1996).

[16] Thom EC. The discomfort index. Weather wise. (1959) 12:57–60.

[17] Silva JAR de Araújo AA Lourenço Júnior JDB dos Santos NDFA Garcia AR de Oliveira RP. Thermal comfort indices of female Murrah buffaloes reared in the Eastern Amazon. Int J Biometeorol. (2015) 59:1261–7. doi: 10.1007/s00484-014-0937-y, PMID: 26041385

[18] R Core Team. (2016). R: A Language Environment for Statistical Computing. R Foundation for Statistical Computing, Vienna. Available at: https://www.r-project.org/ (Accessed August 6, 2022)

[19] Marai IFM Haeeb AAM. Buffalo’s biological functions as affected by heat stress—A review. Livest Sci. (2010) 127:89–109. doi: 10.1016/j.livsci.2009.08.001

[20] Hassan FU Nawaz A Rehman MS Ali MA Dilshad SM Yang C. Prospects of HSP70 as a genetic marker for thermo-tolerance and immuno-modulation in animals under climate change scenario. Anim Nutr. (2019) 5:340–50. doi: 10.1016/j.aninu.2019.06.005, PMID: 31890910 PMC6920399

[21] Das R Sailo L Verma N Bharti P Saikia J Kumar R. Impact of heat stress on health and performance of dairy animals: A review. Vet World. (2016) 9:260. doi: 10.14202/vetworld.2016.260-268, PMID: 27057109 PMC4823286

[22] Lendez PA Cuesta LM Farias MVN Vater AA Ghezzi MD Mota-Rojas D. Alterations in TNF-α and its receptors expression in cows undergoing heat stress. Vet Immunol Immunopathol. (2021) 235:110232. doi: 10.1016/j.vetimm.2021.110232, PMID: 33799007

[23] Ahirwar MK Kataktalwarea MA Pushpadassa HA Jeyakumara S Jashb S Nazara S. Scrotal infrared digital thermography predicts effects of thermal stress on bulalo (*Bubalus bubalis*) sêmen. J Therm Biol. (2018) 78:51–7. doi: 10.1016/j.jtherbio.2018.09.003, PMID: 30509667

[24] Castro SR Rebelo LS Fernandes Junior OS Belo-Reis AS Neves KA Silva WC. Influence of seasonality on the physiological and seminal parameters of buffaloes in the western region of Pará. Pesquisa Veterinária Brasileira. (2021) 40:1048–53.

[25] Kapila N Sharma A Kishore A Sodhi M Tripathi PK Mohanty AK. Impact of heat stress on cellular and transcriptional adaptation of mammary epithelial cells in riverine buffalo (*Bubalus bubalis*). PLoS One. (2016) 11:e0157237. doi: 10.1371/journal.pone.0157237, PMID: 27682256 PMC5040452

[26] Barros DV Silva LKX Kahwage PR Lourenço Júnior JDB Sousa JS Silva AGM. Assessment of surface temperatures of buffalo bulls (*Bubalus bubalis*) raised under tropical conditions using infrared thermography. Arquivo Brasileiro de Medicina Veterinária e Zootecnia. (2016) 68:422–30. doi: 10.1590/1678-4162-8327

[27] Choudhary BB Sirohi S. Sensitivity of buffaloes (*Bubalus bubalis*) to heat stress. J Dairy Res. (2019) 86:399–405. doi: 10.1017/S0022029919000773, PMID: 31787123

[28] Milera M de la López CO Alonso O. Princípios gerados a partir da evolução do manejo em pastoreio para a produção de leite bovino em Cuba Evolução do manejo do pastoreio para a produção leiteira em Cuba. Pastos e Forrajes. (2014) 37:382–91.

[29] Milera MC. Recursos forrajeros herbáceos y arbóreos. Havana: Estación Experimental de Pastos y Forrajes Indio Hatuey (2016).

[30] Almeida JCFD Joset WCL Noronha RDPP Barbosa AVC Lourenço JDB Silva JARD. Behavior of buffalo heifers reared in shaded and unshaded pastures during the dry season on Marajó Island, Pará, Brazil. Acta Scientiarum Anim Sci. (2019) 41:43088. doi: 10.4025/actascianimsci.v41i1.43088

[31] Azevêdo DMMR Alves AA. Bioclimatologia aplicada à produção de bovinos leiteiros nos trópicos. Teresina: Embrapa Meio-Norte (2009). 83 p.

[32] Armstrong DV. Heat stress interaction with shade and cooling. J Dairy Sci. (1994) 77:2044–50. doi: 10.3168/jds.S0022-0302(94)77149-6, PMID: 7929964

[33] Moraes RJ Jr Garcia AR Santos NFA Nahúm BS Lourenço JB Jr Araújo CV. Conforto ambiental de bezerros bubalinos (*Bubalus bubalis* Linnaeus, 1758) em sistemas silvipastoris na Amazônia Oriental. Acta Amazonia. (2010) 40:629–40. doi: 10.1590/S0044-59672010000400001

[34] Pantoja MH Silva JAR Delgado ML Margarido YMM Adami COR Garcia AR. Respostas fisiológicas e adaptabilidade de bubalinos ao clima equatorial amazônico. Revista Acadêmica Ciência Anim. (2018) 16:1–7.

[35] Restrepo EM Rosales RB Estrada MXF Orozco JDC Herrera JER. Es posible enfrentar el cambio climático y producir más leche y carne con sistemas silvopastoriles intensivos. Ceiba. (2016) 54:23–30. doi: 10.5377/ceiba.v54i1.2774

[36] López-Vigoa O Lamela-López L Sánchez-Santana T Olivera-Castro Y García-López R González-Ronquillo M. Influencia de la época del año sobre el valor nutricional de los forrajes, en un sistema silvopastoril. Pastos Forrajes. (2019) 42:57–67.

[37] Galloso-Hernández MA Rodríguez-Estévez V Alvarez-Díaz CA Soca-Pérez M Dublin D Iglesias-Gómez J. Effect of silvopastoral systems in the thermoregulatory and feeding behaviors of water buffaloes under different conditions of heat stress. Front Vet Sci. (2020) 7:393. doi: 10.3389/fvets.2020.00393 32766293 PMC7379419

[38] Santos N Silva J Araújo A Garcia A Beldini T Rodrigues L. Silvopastoral System Mitigates the Thermal Stress and Benefits Water Buffaloes’ Comfort in the Eastern Amazon, Brazil. J Agricult Stud. (2020) 8:193–202. doi: 10.5296/jas.v8i4.17334

[39] Santos N da Silva J de Araújo A Viana R Garcia A Bezerra A. Silvopastoral Systems Contribute to Water Buffalo Welfare and Normal Behavior Pattern Under Eastern Amazon Conditions. J Agricult Stud. (2022) 9:260–71. doi: 10.5296/jas.v9i2.18022

[40] Marques JDA Maggioni D Abrahao JS Guilherme E Bezerra GA Lugao SMB. Comportamento de touros jovens em confinamento alojados isoladamente ou em grupo. Archivos Latino Americano de Produccion Animal. (2005) 13:97–102.

[41] Façanha DAE Chaves DF Morais JHG Vasconcelos AM Costa WP Guilhermino MM. Tendências metodológicas para avaliação da adaptabilidade ao ambiente tropical. Rev Bras Saúde Prod Anim. (2013) 14:91–103. doi: 10.1590/S1519-99402013000100011

[42] Lopes LB Eckstein C Pina DS Carnevalli RA. The influence of trees on the thermal environment and behaviour of grazing heifers in Brazilian Midwest. Trop Anim Health Prod. (2016) 48:755. doi: 10.1007/s11250-016-1021-x, PMID: 26894499

[43] Fundora O Quintana FO Gonzalez ME. Performance and carcass composition in river buffaloes fed a mixture of star grass, natural pastures and native legumes. Cuban J Agricult Sci. (2004) 38:41–4.

[44] Caraballoso A Borroto Á Pérez R. Conducta de búfalos en pastoreo en humedales de Ciego de Ávila, Cuba. Pastos y Forrajes. (2011) 34:211–7.

[45] Castro AC Lourenço Júnior JDB Santos NDFAD Monteiro EMM Aviz MABD Garcia AR. Silvopastoral system in the Amazon region: tool to increase the productive performance of buffaloes. Cienc Rural. (2008) 38:2395–402. doi: 10.1590/S0103-84782008000800050

[46] Silva JARD Araújo AAD Lourenço Júnior JDB Santos NDFAD Garcia AR Nahúm BDS. Conforto térmico de búfalas em sistema silvipastoril na Amazônia Oriental. Pesq Agrop Brasileira. (2011) 46:1364–71. doi: 10.1590/S0100-204X2011001000033

[47] Dash S Chakravarty AK Singh A Shivahre PR Upadhyay A Sah V. Assessment of expected breeding values for fertility traits of Murrah buffaloes under subtropical climate. Vet World. (2015) 8:320. doi: 10.14202/vetworld.2015.320-325, PMID: 27047091 PMC4774837

[48] Frisch JE Vercoe JE. Adaptive and productive features of cattle growth in the tropics: their relevance to buffalo production. Trop Anim Health Prod. (1979) 4:214–22.

[49] Upadhyay R Chaiyabutr N. (2017). Thermal balance in the buffalo species. The buffalo (*bubalus bubalis*) – Production and Research. 1st Edn Sharjah: Bentham Science Publishers, pp. 105–144.

[50] Habeeb AAM El-Tarabany AA Gad AE Atta MA. Negative effects of heat stress on physiological and immunity responses of farm animals. Change. (2018) 16:01–2.

[51] Habeeb AAM Fatma FIT Osman SF. Detection of heat adaptability using heat shock proteins and some hormones in Egyptian buffalo calves. Egyptian J Appl Sci. (2007) 22:28–53.

[52] Khongdee T Sripoon S Vajrabukka C. The effects of high temperature and wallow on physiological responses of swamp buffaloes (*Bubalus bubalis*) during winter season in Thailand. J Therm Biol. (2011) 36:417–21. doi: 10.1016/j.jtherbio.2011.07.006

[53] Savsani HH Padodara RJ Bhadaniya AR Kalariya VA Javia BB Ghodasara SN. Impact of climate on feeding, production and reproduction of animals-A Review. Agric Rev. (2015) 36:3. doi: 10.5958/0976-0741.2015.00003.3

[54] Ahmad M Bhatti JA Abdullah M Javed K Din R Ali M. Effect of different ambient management interventions on milk production and physiological performance of lactating Nili-Ravi buffaloes during hot humid summer. Livest Res Rural Dev. (2017) 29:230.

[55] De Rosa G Grasso F Pacelli C Napolitano F Winckler C. The welfare of dairy buffalo. Ital J Anim Sci. (2009) 8:103–16. doi: 10.4081/ijas.2009.s1.103

[56] Aggarwal A Singh M. Changes in skin and rectal temperature in lactating buffaloes provided with showers and wallowing during hot-dry season. Trop Anim Health Prod. (2008) 40:223–8. doi: 10.1007/s11250-007-9084-3, PMID: 18484125

[57] Pereira AM Vilela RA Titto CG Leme-dos-Santos T Geraldo A Balieiro JC. Thermoregulatory responses of heat acclimatized buffaloes to simulated heat waves. Animals. (2020) 10:756. doi: 10.3390/ani10050756, PMID: 32357524 PMC7277657

[58] Bertoni A Napolitano F Mota-Rojas D Sabia E Álvarez-Macías A Mora-Medina P. Similarities and differences between river buffaloes and cattle: Health, physiological, behavioral and productivity aspects. J Buffalo Sci. (2020) 9:92–109. doi: 10.6000/1927-520X.2020.09.12

[59] Silva WC Araújo LJS Silva LKX Reis ADSB. Lesões de pele diagnósticadas em búfalos (Bubalis bubalis) na região do baixa amazonas. Revista Brasileira de Ciência Veterinária. (2021) 28:27. doi: 10.4322/rbcv.2021.027

[60] Wankar AK Singh G Yadav B. Thermoregulatory and adaptive responses of adult buffaloes (*Bubalus bubalis*) during hyperthermia: Physiological, behavioral, and metabolic approach. Vet World. (2014) 7:825–30. doi: 10.14202/vetworld.2014.825-830

[61] Sharma A Kundu SS Tariq H Mahesh MS Gautam S Singh S. Predicting water intake of lactating riverine buffaloes under tropical climate. Livest Sci. (2016) 191:187–90. doi: 10.1016/j.livsci.2016.08.009

[62] Almeida GLP Pandorfi H Barbosa SBP Pereira DF Guiselini C Almeida GAP. Comportamento, produção e qualidade do leite de vacas Holandês-Gir com climatização no curral. Revista Brasileira de Engenharia Agrícola e Ambiental. (2013) 17:892–9. doi: 10.1590/S1415-43662013000800014

[63] Silva WCD Silva JARD Silva ÉBRD Barbosa AVC Sousa CEL Carvalho KCD. Characterization of Thermal Patterns Using Infrared Thermography and Thermolytic Responses of Cattle Reared in Three Different Systems during the Transition Period in the Eastern Amazon, Brazil. Animals. (2023) 13:2735. doi: 10.3390/ani13172735, PMID: 37685000 PMC10487038

[64] Albright JL Arave CW. The behaviour of cattle. Wallingford: CAB International (1996). 306 p.

[65] De Rosa G Napolitano F Grasso F Bilancione A Spadetta M Pacelli C. Welfare quality®: Welfare Quality®: a pan-European integrated Project buffalo. Ital J Anim Sci. (2007) 2:1360–3.

[66] Degasperi SAR Coimbra CH Pimpão CT. Estudo do comportamento do gado Holandês em sistema de semi-confinamento. Revista Acadêmica Ciência Anim. (2003) 1:41–7. doi: 10.7213/cienciaanimal.v1i4.14973

